# Serum Uric Acid Levels and Risk of Eight Site-Specific Cancers: A Mendelian Randomization Study

**DOI:** 10.3389/fgene.2021.608311

**Published:** 2021-03-09

**Authors:** Minxiao Jiang, Liangliang Ren, Songzan Chen, Gonghui Li

**Affiliations:** ^1^Department of Urology, Sir Run-Run Shaw Hospital College of Medicine, Zhejiang University, Hangzhou, China; ^2^Department of Cardiology, Key Laboratory of Biotherapy of Zhejiang Province, Sir Run-Run Shaw Hospital, School of Medicine, Zhejiang University, Hangzhou, China

**Keywords:** Mendelian randomization, uric acid, cancer, risk, causality

## Abstract

The relationship between serum uric acid (UA) levels and cancer risk remains controversial. Here, a two-sample Mendelian randomization analysis was performed to identify a causal effect of serum UA levels on cancer risk. Twenty-six single nucleotide polymorphisms strongly associated with serum UA levels were screened as genetic variants from large-scale meta-analysis data of a genome-wide association study of 110,347 European individuals. Genetic associations with eight common site-specific cancers were subsequently explored. A total of six Mendelian randomization methods were used to estimate the potential effect of serum UA levels on cancer risk, including random effects inverse variance weighting, fix effects inverse variance weighting, MR-Egger, median weighting, mode weighting, and simple mode analysis. Our primary random effects inverse variance weighted analysis revealed that no significant associations with cancers was found (all *p* > 0.05). Sensitivity analyses and additional analyses also showed similar pooled results. In conclusion, no significant causality between serum UA levels and cancer risk was evidenced.

## Introduction

Uric acid (UA) is a byproduct of purine metabolism, with both endogenous and exogenous purines degraded to UA by xanthine oxidase (Benn et al., 2018). Serum UA homeostasis is maintained *via* its production and excretion (Maiuolo et al., 2016), the latter in humans being primarily renal and hepatic (Su et al., 2020). Purine-rich diets, alcohol consumption, obesity, and hypertension are considered to be risk factors that lead to elevated serum UA, in turn resulting in hyperuricemia and even gout (Roddy and Choi, 2014; Li et al., 2020). Hyperuricemia is a common chronic illness defined by a serum UA level >7.0 mg/dl among men and >5.7 mg/dl among women. The incidence of hyperuricemia in the United States is 20.2% in men and 20.0% in women (Chen-Xu et al., 2019).

Previous studies have reported UA levels to be associated with the incidence of diabetes, cardiovascular disease, kidney disease, and malignancies (Weiner et al., 2008; Battelli et al., 2016; Wang et al., 2018; Borghi et al., 2020). The precise mechanistic role UA plays in the occurrence of malignancies, however, remains unclear. Conventional observational studies have reported that higher serum UA levels are protective against cancer (Horsfall et al., 2014; Taghizadeh et al., 2014), while other studies reported higher serum UA levels to increase the risk of a number of malignancies (Strasak et al., 2007; Wang et al., 2015). As observational studies are frequently subject to confounding and a variety of biases, it is difficult to determine whether any causality between serum UA levels and cancer risk exists.

The randomized controlled trial is the gold standard for demonstrating epidemiological causality between exposures and outcomes (Klungel et al., 2004). However, the cost of such trials is high and their strict criteria also produce biases, thus limiting the robustness of results (Evans and Davey Smith, 2015). Mendelian randomization (MR) is a relatively novel and effective analytical method which can reveal causality between exposures and outcomes by considering genetic variants as instrumental variables (Smith and Ebrahim, 2003). Given that genetic variants are randomly distributed, determined at conception, and not associated with other confounders, MR reduces confounding and, to an extent, overcomes reverse causality bias (Emdin et al., 2017).

The role of UA in the pathogenesis of malignancies remains unclear. Here, we designed a two-sample MR study to analyze summary genetic data for the purposes of investigating any potential causal associations of genetically-proxied UA levels and the incidence of eight distinct malignancies.

## Materials and Methods

### Study Design

**Figure 1:**
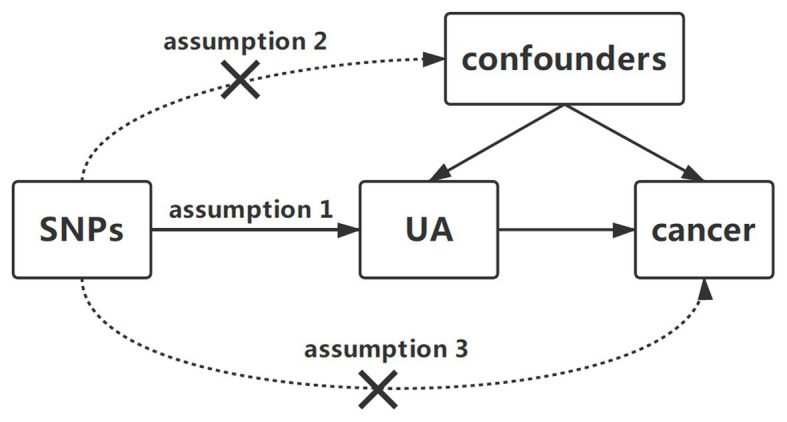
Diagram of two-sample Mendelian randomization analysis of serum uric acid levels and cancer risk. Three key assumptions in the Mendelian randomization analysis are as follows: (1) the SNPs should be related to serum UA levels, (2) the SNPs should be independent of confounders, and (3) the SNPs could affect cancer only by UA. SNP, single-nucleotide polymorphism; UA, uric acid.

To identify the potential effect of serum UA levels on cancer risk, we designed a two-sample MR study. Single nucleotide polymorphisms (SNPs) for serum UA levels were selected as instrumental variables from previously published genome-wide association study (GWAS) analyses. Three key assumptions were to be satisfied: first, the SNPs should have been associated with serum UA levels; second, the chosen SNPs should have been independent of confounders; and third, the SNPs should have affected cancer only *via* UA concentrations and could not have a direct correlation (Figure 1; Little, 2018).

### Exposure Measure

We systematically extracted significant genome-wide SNPs related to serum UA levels from a large-scale GWAS meta-analysis of 110,347 European individuals (49,825 women and 60,522 men). The average age of the participants was 52.12 years. The GWAS data were obtained from the Global Urate Genetics Consortium (GUGC; Table 1; Köttgen et al., 2013). A total of 26 SNPs passed our *p*-value threshold of 5 × 10^−8^, detailing a 7.0% phenotypic variance in serum UA levels. These genetic variants were pruned for linkage disequilibrium using LD-link (https://ldlink.nci.nih.gov/) with an r^2^ threshold of 0.01 (Junqueira et al., 2017). After LD pruning, 26 SNPs remained as genetic instrumental variables to proxy serum UA levels. The average values of serum UA concentrations in these studies were recorded and ranged from 3.9 to 6.1 mg/dl (standard deviation (SD): 0.92–1.68 mg/dl). In addition, the strength of each SNP was evaluated by F-statistic values and the instrument with an F-statistic value larger than 10 was regarded as having strong potential to predict UA levels (Lawlor et al., 2008).

**Table 1:** Characteristics of exposure dataset and outcome datasets.

Phenotype	Consortium	Sample size	Cases	Controls	Ethnicity	References
Serum uric acid	GUGC	110,347			European	Köttgen et al. (2013)
Bladder cancer	UK biobank	361,194	1,367	359,827	European	The Neale lab (2018)
Breast cancer	BCAC	123,509	62,533	60,976	European	Michailidou et al. (2015)
Colorectal cancer	UK biobank	408,458	4,562	382,756	European	Zhou et al. (2018)
Lung cancer	UK biobank	361,194	329	360,865	European	The Neale lab (2018)
Prostate cancer	PRACTICAL	140,254	79,148	61,106	European	Schumacher et al. (2018)
Renal cell cancer	UK biobank	361,194	727	360,467	European	The Neale lab (2018)
Skin cancer	UK biobank	300,791	9,950	290,841	European	Watanabe et al. (2019)
Thyroid cancer	UK biobank	407,757	358	407,399	European	Zhou et al. (2018)

### Outcome Measure

Data from eight, large-scale meta-analyses of GWASs studying eight common cancers were used to explore the association of genetically-proxied serum UA levels with risk of malignancy incidence rates; namely bladder, breast, colorectal, lung, prostate, renal cell, skin, and thyroid cancers. The breast cancer outcome dataset was composed of summary genetic data obtained from the Breast Cancer Association Consortium (BCAC) and consisted of a meta-analysis of 11 GWASs (15,748 cases, 18,084 controls) in addition to 41 studies (46,785 cases, 42,892 controls) genotyped on the iCOGs custom array (Michailidou et al., 2015). The prostate cancer dataset consisted of 79,148 cases and 61,106 controls and was obtained from the Prostate Cancer Association Group to Investigate Cancer Associated Alterations in the Genome (PRACTICAL; Schumacher et al., 2018). The colorectal and thyroid cancer datasets of GWAS meta-analysis data were obtained from the UK Biobank. The colorectal cancer dataset included 4,562 cases and 382,756 controls while the thyroid cancer dataset included 358 cases and 407,399 controls (Zhou et al., 2018). The skin cancer dataset included 9,950 cases and 290,841 controls; this GWAS meta-analysis was performed by the UK Biobank (Watanabe et al., 2019). Bladder (1,367 cases, 359,827 controls), lung (329 cases, 360,865 controls) and renal cell (727 cases, 360,467 controls) cancer datasets were downloaded from the Neale Lab. All aforementioned GWAS meta-analyses only evaluated participants of European descent (Table 1).

### Statistical Analysis

In this study, we used five different methods of MR analysis to evaluate the causal effect of serum UA levels on cancer risk. Here, the random-effects inverse variance weighted (IVW) method was used as the primary analysis. The Wald estimator was used to calculate the ratio of the SNP-outcome estimate over the SNP-exposure estimate, while the Delta method was employed to calculate the standard errors (Teumer, 2018). The overall estimate was subsequently obtained by pooling the Wald ratio estimates of each SNP weighted by inverse variances of the SNP-outcome associations (Little, 2018). As this method assumes that the intercept is constrained to the origin [0,0], the presence of horizontal pleiotropy makes this method susceptible to bias. To supplement calculations, we used MR-Egger regression, where intercept and slope represent the average horizontal pleiotropy and the pleiotropy-adjusted MR estimate, respectively. In addition, we utilized weighted median analysis to estimate the effects of all MR estimates that every individual instrument was weighted equally to the inverse of the standard error. Weighted median analysis served as an important method of estimating the causal effect if over 50% of SNPs met the “no horizontal pleiotropy” assumption (Burgess et al., 2017). Finally, weighted mode and simple mode analyses were used to estimate the causal effect (Hemani et al., 2018a).

For the individual variants in the genetic instrumental variable model for serum UA levels, we examined whether some SNPs had a significantly independent influence on results *via* leave-one-out sensitivity analysis. The remaining estimate effect was shown when one SNP was excluded (Hemani et al., 2018b). Cochran’s Q statistics were used to estimate the level of heterogeneity.

In addition, we searched traits associated with all 26 SNPs on the PhenoScanner website.^1^ After excluding SNPs that were not exclusively associated with UA levels, MR analysis was repeated for the purposes of improving result robustness and deal with potential horizontal pleiotropy.

All statistical analyses in this paper were performed using R software (version 4.0.2; http://www.rproject.org) with the “TwoSampleMR” package (version 0.5.4). Results were considered to show strong evidence of an association between serum UA levels and cancer incidence if they surpassed a stringent Bonferroni-corrected *p*-value threshold of 6.25 × 10^−3^ (0.05/8 cancer outcomes).

## Results

Here, 26 SNPs strongly related to serum UA levels were extracted from a GWAS meta-analysis based on GUGC data (*p* < 5 × 10^−8^; Supplementary Table S1). No linkage disequilibrium (r^2^ < 0.01) was observed. The minimum F-statistic value of these 26 SNPs was 30.05, suggesting that they were sufficiently effective in this study. All SNPs could thus be used to identify the potential effect of serum UA levels on cancer risk. Scatter plots were shown in the supplementary materials (Supplementary Figures S1–S8a).

Using a genetic instrumental variable for serum UA levels consisting of 26 SNPs, we estimated the association of serum UA levels against the incidence of eight distinct cancers *via* MR analysis. Associations with individual cancers were described below.

### Effect of Uric Acid on Cancers

**Figure 2:**
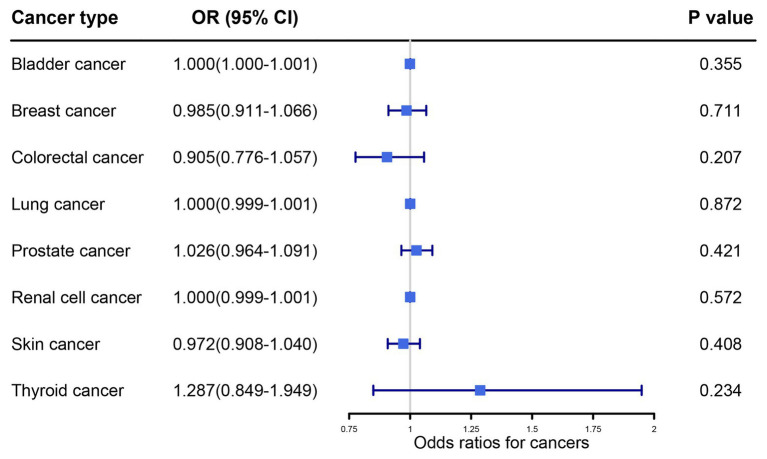
Primary results of the causal associations between serum uric acid levels and cancer risk by random effects inverse variance weighted method. OR, odds ratio; IVW, inverse variance weighted; CI, confidence interval.

The main MR results detailing the influence of UA levels on cancers were obtained using random-effects IVW methodology (Figure 2). Our primary results did not reveal any association between serum UA levels and the risk of any other cancer type (all *p* > 0.05).

The MR estimates of UA levels on cancer risk obtained using other sensitivity MR approaches are shown in the supplementary materials (Supplementary Table S2). Consistent results using MR-Egger, weighted median, weighted mode, and simple mode analyses were not obtained regarding the risk of all cancer types. In addition, leave-one-out sensitivity analysis was performed and revealed that no SNP could independently drive MR analysis results for most of cancers (Supplementary Figures S1–S8b). However, it was observed that rs12498742 had a significant effect on the results for colorectal and skin cancers. Therefore, we performed analysis based on SNPs excluding the rs12498742. Similarly, no causal effect was observed in the results after Bonferroni correction (Supplementary Figure S9).

### Other Analysis

The results of MR-Egger regression for the assessment of pleiotropy are listed in Supplementary Table S3, suggesting that the non-pleiotropy assumption was satisfied in all of the aforementioned MR methods for most cancers. However, we detected significant pleiotropy when testing for a causal effect of UA on skin cancer risk (intercept = −0.01, *p* = 0.03). In addition, some evidence of heterogeneity was also found using Cochran’s Q statistics for some cancers, including breast, colorectal and prostate cancer (Supplementary Table S4). To deal with heterogeneity and potential horizontal pleiotropy, all 26 SNPs were searched on the PhenoScanner website and six SNPs were found to be exclusively associated with serum UA levels (Supplementary Table S5). The entire analysis was subsequently repeated using these six SNPs as instruments. Similarly, no significant causal relationship was observed between serum UA levels and the eight site-specific cancers in question (Supplementary Table S6). These results strongly suggested that the observed associations were not biased by pleiotropic effects (Supplementary Table S3). Results of heterogeneity testing also revealed a significant decrease of heterogeneity after excluding SNPs associated with the phenomenon, apart from serum UA levels (Supplementary Table S4).

## Discussion

This study explored the relationship between serum UA levels and cancer risk *via* a two-sample MR analysis and did not identify strong evidence supporting causality between serum UA levels and cancer risk, including that of bladder, breast, colorectal, lung, prostate, renal cell, skin, and thyroid cancer. The sensitivity analyses and other analyses supported these findings.

Ames et al. first reported that serum UA was an excellent scavenger of singlet oxygen and hydroxyl radicals, and could be a protective factor against cancer in humans (Ames et al., 1981). Evidence for the antioxidant function of UA has continued to increase over the recent decades (Peden et al., 1990; Becker, 1993; Liu et al., 2019). Itahana et al. demonstrated that SLC2A9 was a key downstream target of p53, already well known as a typical UA transporter. This pathway was found to be protective from ROS-induced damage and cancer pathogenesis in humans (Itahana et al., 2015). In contrast, UA levels are also regarded to be a risk factor for cancer due to its function in inducing chronic inflammation and increasing ROS production (Mi et al., 2020). Chronic inflammation and tissue infiltration by neutrophils, macrophages and monocytes (Grainger et al., 2013; Weigt et al., 2017), in turn, promote carcinogenesis (Fini et al., 2012; Braga et al., 2017; Ahechu et al., 2018). Due to the complex roles UA plays in cancer occurrence, associations remain unconfirmed in previous epidemiological literature (Strasak et al., 2007; Dziaman et al., 2014; Horsfall et al., 2014; Szkandera et al., 2015; Battelli et al., 2016; Mi et al., 2020).

A recently published prospective population-based study demonstrated associations between serum UA levels and the risks of common cancers (Kuhn et al., 2017). Consistent with our findings, serum UA levels were reported not to be associated with risks of lung, prostate, and colorectal cancer. In addition, higher serum UA levels were associated with lower breast cancer risk. The implications of these results, however, were limited due to the number of cases in this cohort study (lung cancer, *n* = 195; colorectal cancer, *n* = 256; breast cancer, *n* = 627; prostate cancer, *n* = 554). One MR study also revealed causal relationships between serum UA levels and cancer risks. The study was based on the Copenhagen General Population Study and selected rs7442295 (gene: SLC2A9, position: chr4:9964756) as the instrument for serum UA level representation. Results revealed causal relationships between high serum UA levels, high cancer incidence and high all-cause mortality, contrary to our findings. Possible explanations of such a paradoxical finding were that this study only used rs7442295 as the instrument which could only explain 2% of the variation in serum UA levels, and SLC2A9 was expressed differently in various organs, thus there might some biases in that study (Kobylecki et al., 2017). Li et al. additionally reviewed the relationship between serum UA levels and multiple health outcomes recently. Similarly, the review did not identify convincing evidence supporting a clear influence of serum UA levels on cancer outcomes (Li et al., 2017).

Here, we designed an MR study to investigate potential causality between serum UA levels and malignancy risk. To meet the first assumption, the genetic instrument chosen in the MR study should be strongly associated with serum UA levels. A previous study has reported that almost 40–80% of variation of serum UA levels could be explained by genetic factors (Krishnan et al., 2012), while the strength of genetic instruments used in MR studies was still small and accounted for only 7% of serum UA variance. The power of genetic instruments to detect causal associations with serum UA levels was thereby limited. Nevertheless, all SNPs chosen in this study passed our *p*-value threshold of 5 × 10^−8^ and F-statistics values threshold of 10, indicating all instruments were sufficiently effective. Sensitivity analyses, including five MR methods and pleiotropy analyses, were subsequently carried out to evaluate for potential violation of the second assumption. Results revealed some evidence for the existence of horizontal pleiotropy and heterogeneity in the analysis by using 26 SNPs, indicting this assumption may be violated. After exclusion of the SNPs not exclusively associated with serum UA levels, we found the heterogeneity and pleiotropy decreased significantly and the results remained unchanged, which proved the robustness of the results. In addition, no SNP was found to be associated directly with cancers. Therefore, the likelihood of biases in this paper is low.

This study has several advantages. First, almost all prior studies were observational and incorporated a limited quantity of patients, thus likely causing observation bias and increasing the risk of confounding. Furthermore, few studies have demonstrated potential causality between serum UA levels and cancer risk. Our study used a novel method, MR analysis, to assess any potential causal relationship, thereby minimizing confounding and overcoming reverse causality. The two-sample MR method also allowed us to integrate several independent GWAS datasets with large sample sizes and yield more precise results. To the best of our knowledge, this study was the largest such MR analysis focused on the relationship between serum UA levels and malignancy. Moreover, six different MR methods were employed in this study, thus increasing result robustness.

Our study, however, was not without limitations. First, the proportions of cases for some site-specific cancers were low, and it might result in a low precision of the estimates (Zhou et al., 2018). Second, data for most cancers was downloaded from the UK Biobank. The individuals in the UK Biobank are healthier than the general population, and we cannot rule out the “healthy volunteer” selection bias (Fry et al., 2017). Third, due to a lack of detailed information in the datasets, we were unable to conduct more refined analyses (e.g., stratification analysis). In addition, the GWAS datasets in this study only contained data from European individuals. Our findings may thus not be applicable to other races. Future studies should evaluate patients from different ethnicities and in wider age ranges.

## Conclusion

In conclusion, we did not find any consistently strong evidence supporting causality between serum UA levels and cancer risk. However, the potential causal role of serum UA levels in the risk of malignancy warrants further investigation by studying a greater number of cancer types and employing larger MR analyses.

## Data Availability Statement

The original contributions presented in the study are included in the article/Supplementary Material, further inquiries can be directed to the corresponding author.

## Ethics Statement

Written informed consent was obtained from the individual(s) for the publication of any potentially identifiable images or data included in this article.

## Author Contributions

MJ and GL designed the study. MJ and LR assembled and analyzed the data. LR and SC visualized the data. MJ, LR, and SC wrote the paper. All authors contributed to the article and approved the submitted version.

### Conflict of Interest

The authors declare that the research was conducted in the absence of any commercial or financial relationships that could be construed as a potential conflict of interest.

## Footnotes

^1^http://www.phenoscanner.medschl.cam.ac.uk/phenoscanner

## References

[ref1] Ahechu P. Zozaya G. Marti P. Hernandez-Lizoain J. L. Baixauli J. Unamuno X.. (2018). NLRP3 Inflammasome: a possible link between obesity-associated low-grade chronic inflammation and colorectal cancer development. Front. Immunol. 9:2918. 10.3389/fimmu.2018.02918, PMID: 30619282 PMC6297839

[ref2] Ames B. N. Cathcart R. Schwiers E. Hochstein P. (1981). Uric acid provides an antioxidant defense in humans against oxidant- and radical-caused aging and cancer: a hypothesis. Proc. Natl. Acad. Sci. U. S. A. 78, 6858–6862. 10.1073/pnas.78.11.6858, PMID: 6947260 PMC349151

[ref3] Battelli M. G. Polito L. Bortolotti M. Bolognesi A. (2016). Xanthine oxidoreductase in cancer: more than a differentiation marker. Cancer Med. 5, 546–557. 10.1002/cam4.601, PMID: 26687331 PMC4799950

[ref4] Becker B. F. (1993). Towards the physiological function of uric acid. Free Radic. Biol. Med. 14, 615–631. 10.1016/0891-5849(93)90143-I, PMID: 8325534

[ref5] Benn C. L. Dua P. Gurrell R. Loudon P. Pike A. Storer R. I.. (2018). Physiology of hyperuricemia and urate-lowering treatments. Front. Med. 5:160. 10.3389/fmed.2018.00160, PMID: 29904633 PMC5990632

[ref6] Borghi C. Agabiti-Rosei E. Johnson R. J. Kielstein J. T. Lurbe E. Mancia G.. (2020). Hyperuricaemia and gout in cardiovascular, metabolic and kidney disease. Eur. J. Intern. Med. 80, 1–11. 10.1016/j.ejim.2020.07.006, PMID: 32739239

[ref7] Braga T. T. Forni M. F. Correa-Costa M. Ramos R. N. Barbuto J. A. Branco P.. (2017). Soluble uric acid activates the NLRP3 Inflammasome. Sci. Rep. 7:39884. 10.1038/srep39884, PMID: 28084303 PMC5233987

[ref8] Burgess S. Bowden J. Fall T. Ingelsson E. Thompson S. G. (2017). Sensitivity analyses for robust causal inference from Mendelian randomization analyses with multiple genetic variants. Epidemiology 28, 30–42. 10.1097/EDE.0000000000000559, PMID: 27749700 PMC5133381

[ref9] Chen-Xu M. Yokose C. Rai S. K. Pillinger M. H. Choi H. K. (2019). Contemporary prevalence of gout and hyperuricemia in the United States and decadal trends: the national health and nutrition examination survey, 2007-2016. Arthritis Rheum. 71, 991–999. 10.1002/art.40807, PMID: 30618180 PMC6536335

[ref10] Dziaman T. Banaszkiewicz Z. Roszkowski K. Gackowski D. Wisniewska E. Rozalski R.. (2014). 8-Oxo-7,8-dihydroguanine and uric acid as efficient predictors of survival in colon cancer patients. Int. J. Cancer 134, 376–383. 10.1002/ijc.28374, PMID: 23832862

[ref11] Emdin C. A. Khera A. V. Kathiresan S. (2017). Mendelian randomization. JAMA 318, 1925–1926. 10.1001/jama.2017.17219, PMID: 29164242

[ref12] Evans D. M. Davey Smith G. (2015). Mendelian randomization: new applications in the coming age of hypothesis-free causality. Annu. Rev. Genomics Hum. Genet. 16, 327–350. 10.1146/annurev-genom-090314-050016, PMID: 25939054

[ref13] Fini M. A. Elias A. Johnson R. J. Wright R. M. (2012). Contribution of uric acid to cancer risk, recurrence, and mortality. Clin. Transl. Med. 1:16. 10.1186/2001-1326-1-16, PMID: 23369448 PMC3560981

[ref14] Fry A. Littlejohns T. J. Sudlow C. Doherty N. Adamska L. Sprosen T.. (2017). Comparison of sociodemographic and health-related characteristics of UK biobank participants with those of the general population. Am. J. Epidemiol. 186, 1026–1034. 10.1093/aje/kwx246, PMID: 28641372 PMC5860371

[ref15] Grainger R. McLaughlin R. J. Harrison A. A. Harper J. L. (2013). Hyperuricaemia elevates circulating CCL2 levels and primes monocyte trafficking in subjects with inter-critical gout. Rheumatology 52, 1018–1021. 10.1093/rheumatology/kes326, PMID: 23204548

[ref16] Hemani G. Bowden J. Davey Smith G. (2018a). Evaluating the potential role of pleiotropy in Mendelian randomization studies. Hum. Mol. Genet. 27, R195–R208. 10.1093/hmg/ddy163, PMID: 29771313 PMC6061876

[ref17] Hemani G. Zheng J. Elsworth B. Wade K. H. Haberland V. Baird D.. (2018b). The MR-base platform supports systematic causal inference across the human phenome. elife 7:e34408. 10.7554/eLife.34408, PMID: 29846171 PMC5976434

[ref18] Horsfall L. J. Nazareth I. Petersen I. (2014). Serum uric acid and the risk of respiratory disease: a population-based cohort study. Thorax 69, 1021–1026. 10.1136/thoraxjnl-2014-205271, PMID: 24904021 PMC4215274

[ref19] Itahana Y. Han R. Barbier S. Lei Z. Rozen S. Itahana K. (2015). The uric acid transporter SLC2A9 is a direct target gene of the tumor suppressor p53 contributing to antioxidant defense. Oncogene 34, 1799–1810. 10.1038/onc.2014.119, PMID: 24858040

[ref20] Junqueira S. C. Dos Santos Coelho I. Lieberknecht V. Cunha M. P. Calixto J. B. Rodrigues A. L. S.. (2017). Inosine, an endogenous purine nucleoside, suppresses immune responses and protects mice from experimental autoimmune encephalomyelitis: a role for A2A adenosine receptor. Mol. Neurobiol. 54, 3271–3285. 10.1007/s12035-016-9893-3, PMID: 27130268

[ref21] Klungel O. H. Martens E. P. Psaty B. M. Grobbee D. E. Sullivan S. D. Stricker B. H.. (2004). Methods to assess intended effects of drug treatment in observational studies are reviewed. J. Clin. Epidemiol. 57, 1223–1231. 10.1016/j.jclinepi.2004.03.011, PMID: 15617947

[ref22] Kobylecki C. J. Afzal S. Nordestgaard B. G. (2017). Plasma urate, cancer incidence, and all-cause mortality: a Mendelian randomization study. Clin. Chem. 63, 1151–1160. 10.1373/clinchem.2016.268185, PMID: 28428355

[ref23] Köttgen A. Albrecht E. Teumer A. Vitart V. Krumsiek J. Hundertmark C.. (2013). Genome-wide association analyses identify 18 new loci associated with serum urate concentrations. Nat. Genet. 45, 145–154. 10.1038/ng.2500, PMID: 23263486 PMC3663712

[ref24] Krishnan E. Lessov-Schlaggar C. N. Krasnow R. E. Swan G. E. (2012). Nature versus nurture in gout: a twin study. Am. J. Med. 125, 499–504. 10.1016/j.amjmed.2011.11.010, PMID: 22365026

[ref25] Kuhn T. Sookthai D. Graf M. E. Schubel R. Freisling H. Johnson T.. (2017). ALbumin, bilirubin, uric acid and cancer risk: results from a prospective population-based study. Br. J. Cancer 117, 1572–1579. 10.1038/bjc.2017.313, PMID: 28898231 PMC5680462

[ref26] Lawlor D. A. Harbord R. M. Sterne J. A. C. Timpson N. Smith G. D. (2008). Mendelian randomization: using genes as instruments for making causal inferences in epidemiology. Stat. Med. 27, 1133–1163. 10.1002/sim.3034, PMID: 17886233

[ref27] Li X. Meng X. Timofeeva M. Tzoulaki I. Tsilidis K. K. Ioannidis J. P.. (2017). Serum uric acid levels and multiple health outcomes: umbrella review of evidence from observational studies, randomised controlled trials, and Mendelian randomisation studies. BMJ 357:j2376. 10.1136/bmj.j2376, PMID: 28592419 PMC5461476

[ref28] Li L. Zhang Y. Zeng C. (2020). Update on the epidemiology, genetics, and therapeutic options of hyperuricemia. Am. J. Transl. Res. 12, 3167–3181. PMID: 32774692 PMC7407685

[ref29] Little M. (2018). Mendelian randomization: methods for using genetic variants in causal estimation. J. Roy. Stat. Soc. Stat. Soc. 181, 549–550. 10.1111/rssa.12343

[ref30] Liu D. Q. Yun Y. Yang D. C. Hu X. Y. Dong X. X. Zhang N.. (2019). What is the biological function of uric acid? An antioxidant for neural protection or a biomarker for cell death. Dis. Markers 2019, 1–9. 10.1155/2019/4081962 PMC6348815 30733836

[ref31] Maiuolo J. Oppedisano F. Gratteri S. Muscoli C. Mollace V. (2016). Regulation of uric acid metabolism and excretion. Int. J. Cardiol. 213, 8–14. 10.1016/j.ijcard.2015.08.109, PMID: 26316329

[ref32] Mi S. Gong L. Sui Z. (2020). Friend or foe? An unrecognized role of uric acid in cancer development and the potential anticancer effects of uric acid-lowering drugs. J. Cancer 11, 5236–5244. 10.7150/jca.46200, PMID: 32742469 PMC7378935

[ref33] Michailidou K. Beesley J. Lindstrom S. Canisius S. Dennis J. Lush M. J.. (2015). Genome-wide association analysis of more than 120,000 individuals identifies 15 new susceptibility loci for breast cancer. Nat. Genet. 47, 373–U127. 10.1038/ng.3242, PMID: 25751625 PMC4549775

[ref34] Peden D. B. Hohman R. Brown M. E. Mason R. T. Berkebile C. Fales H. M.. (1990). Uric-acid is a major antioxidant in human nasal airway secretions. Proc. Natl. Acad. Sci. U. S. A. 87, 7638–7642. 10.1073/pnas.87.19.7638 2217195 PMC54803

[ref35] Roddy E. Choi H. K. (2014). Epidemiology of gout. Rheum. Dis. Clin. N. Am. 40, 155–175. 10.1016/j.rdc.2014.01.001, PMID: 24703341 PMC4119792

[ref36] Schumacher F. R. Al Olama A. A. Berndt S. I. Benlloch S. Ahmed M. Saunders E. J.. (2018). Association analyses of more than 140,000 men identify 63 new prostate cancer susceptibility loci. Nat. Genet. 50, 928–936. 10.1038/s41588-018-0142-8, PMID: 29892016 PMC6568012

[ref37] Smith G. D. Ebrahim S. (2003). Mendelian randomization': can genetic epidemiology contribute to understanding environmental determinants of disease? Int. J. Epidemiol. 32, 1–22. 10.1093/ije/dyg070, PMID: 12689998

[ref38] Strasak A. M. Rapp K. Hilbe W. Oberaigner W. Ruttmann E. Concin H.. (2007). The role of serum uric acid as an antioxidant protecting against cancer: prospective study in more than 28000 older Austrian women. Ann. Oncol. 18, 1893–1897. 10.1093/annonc/mdm338, PMID: 17785768

[ref39] Su H. Y. Yang C. Liang D. Liu H. F. (2020). Research advances in the mechanisms of hyperuricemia-induced renal injury. Biomed. Res. Int. 2020, 1–12. 10.1155/2020/5817348 PMC7336201 32685502

[ref40] Szkandera J. Gerger A. Liegl-Atzwanger B. Stotz M. Samonigg H. Ploner F.. (2015). Uric acid levels in blood are associated with clinical outcome in soft-tissue sarcoma patients. Clin. Chem. Lab. Med. 53, 493–497. 10.1515/cclm-2014-0486, PMID: 25324451

[ref41] Taghizadeh N. Vonk J. M. Boezen H. M. (2014). Serum uric acid levels and cancer mortality risk among males in a large general population-based cohort study. Cancer Causes Control 25, 1075–1080. 10.1007/s10552-014-0408-0, PMID: 24906474 PMC4082647

[ref42] Teumer A. (2018). Common methods for performing Mendelian randomization. Front. Cardiovasc. Med. 5:51. 10.3389/fcvm.2018.00051, PMID: 29892602 PMC5985452

[ref500] The Neale lab (2018). http://www.nealelab.is/uk-biobank/ (Accessed August 15, 2020).

[ref43] Wang W. Xu D. Wang B. Yan S. Wang X. Yin Y.. (2015). Increased risk of cancer in relation to gout: a review of three prospective cohort studies with 50,358 subjects. Mediat. Inflamm. 2015:680853. 10.1155/2015/680853, PMID: 26504360 PMC4609488

[ref44] Wang H. Zhang H. Sun L. Guo W. (2018). Roles of hyperuricemia in metabolic syndrome and cardiac-kidney-vascular system diseases. Am. J. Transl. Res. 10, 2749–2763. PMID: 30323864 PMC6176241

[ref45] Watanabe K. Stringer S. Frei O. Umicevic Mirkov M. de Leeuw C. Polderman T. J. C.. (2019). A global overview of pleiotropy and genetic architecture in complex traits. Nat. Genet. 51, 1339–1348. 10.1038/s41588-019-0481-0, PMID: 31427789

[ref46] Weigt S. S. Palchevskiy V. Belperio J. A. (2017). Inflammasomes and IL-1 biology in the pathogenesis of allograft dysfunction. J. Clin. Invest. 127, 2022–2029. 10.1172/JCI93537, PMID: 28569730 PMC5451233

[ref47] Weiner D. E. Tighiouart H. Elsayed E. F. Griffith J. L. Salem D. N. Levey A. S. (2008). Uric acid and incident kidney disease in the community. J. Am. Soc. Nephrol. 19, 1204–1211. 10.1681/ASN.2007101075, PMID: 18337481 PMC2396939

[ref48] Zhou W. Nielsen J. B. Fritsche L. G. Dey R. Gabrielsen M. E. Wolford B. N.. (2018). Efficiently controlling for case-control imbalance and sample relatedness in large-scale genetic association studies. Nat. Genet. 50, 1335–1341. 10.1038/s41588-018-0184-y, PMID: 30104761 PMC6119127

