# Supplementary material for: Serum Uric Acid Levels and Risk of Eight Site-Specific Cancers: A Mendelian Randomization Study
Source: Front Genet. 2021 Mar 9;12:608311. doi: 10.3389/fgene.2021.608311 (PMC7985250; doi:10.3389/fgene.2021.608311)
Supplement: Supplementary file 1 [file Data_Sheet_1.pdf]

Supplementary Material

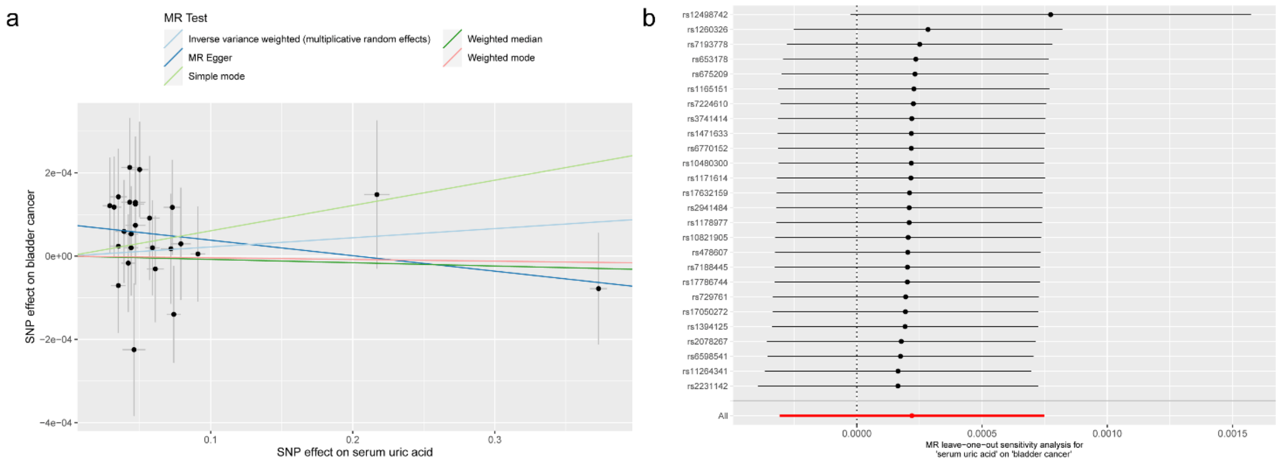

Supplementary Figure 1. Scatter plot and leave-one-out plot of the estimated effect of serum uric acid levels on risk of bladder cancer. (a) scatter plot; (b) leave-one-out plot.

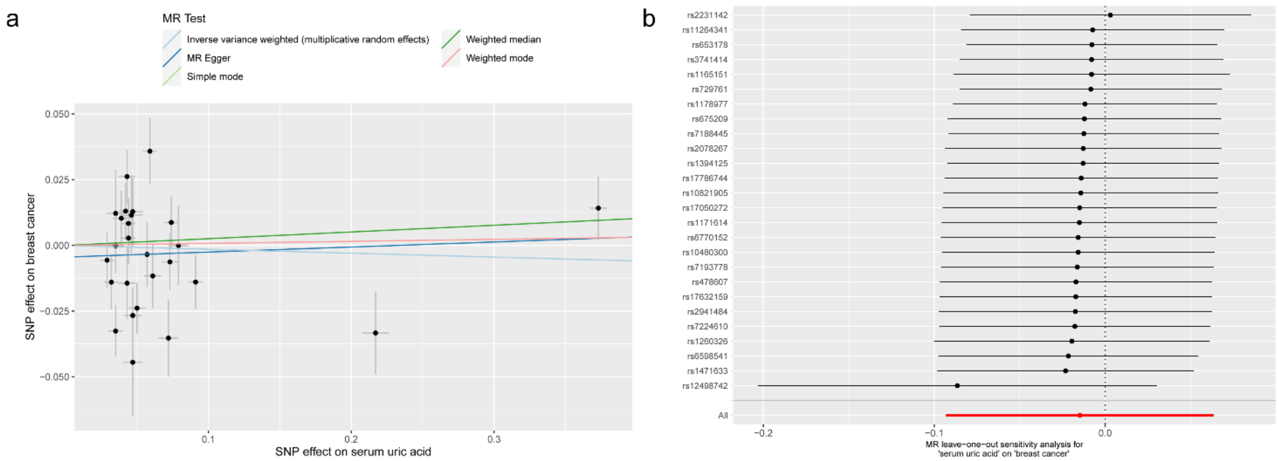

Supplementary Figure 2. Scatter plot and leave-one-out plot of the estimated effect of serum uric acid levels on risk of breast cancer. (a) scatter plot; (b) leave-one-out plot.

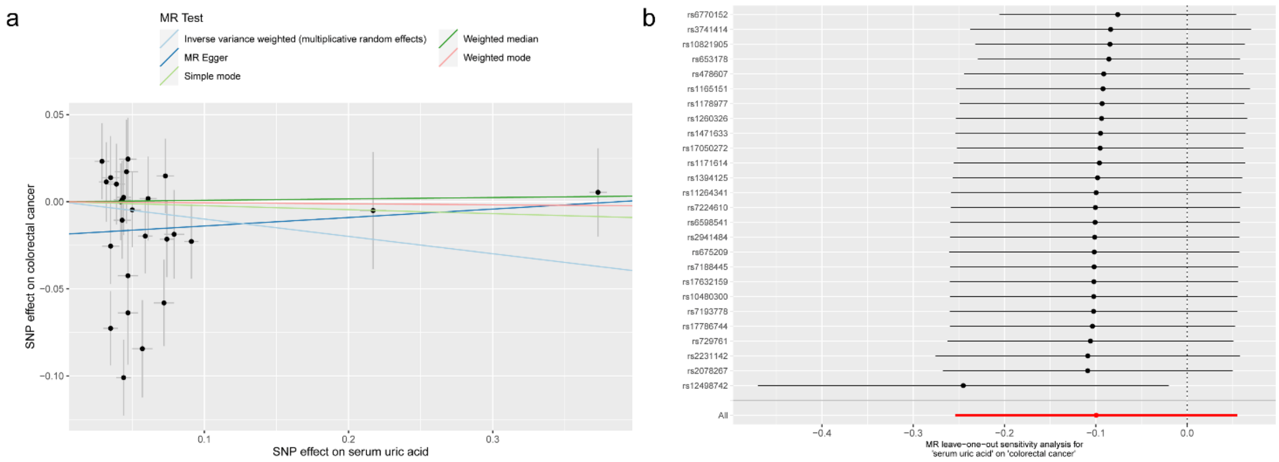

Supplementary Figure 3. Scatter plot and leave-one-out plot of the estimated effect of serum uric acid levels on risk of colorectal cancer. (a) scatter plot; (b) leave-one-out plot.

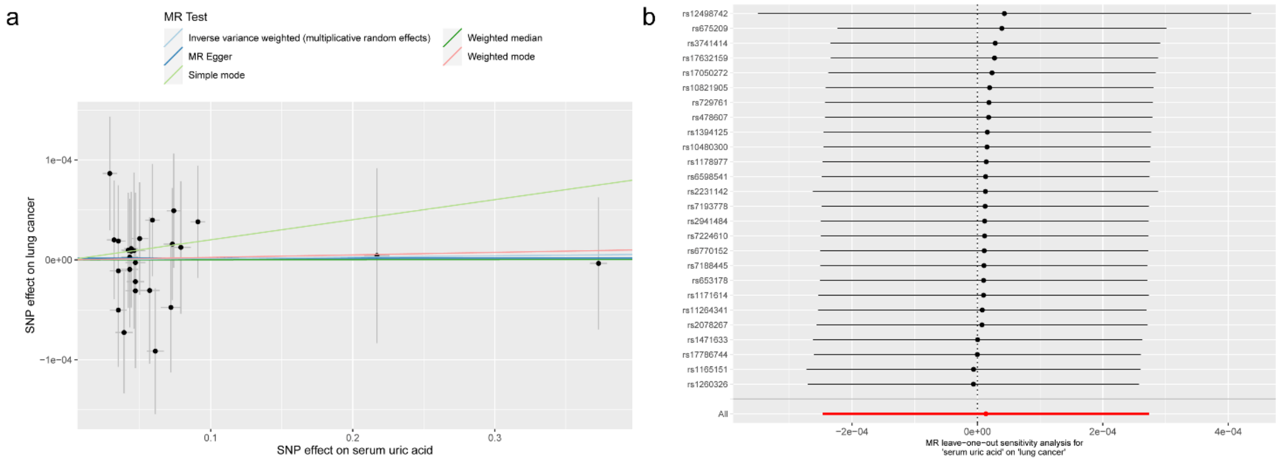

Supplementary Figure 4. Scatter plot and leave-one-out plot of the estimated effect of serum uric acid levels on risk of lung cancer. (a) scatter plot; (b) leave-one-out plot.

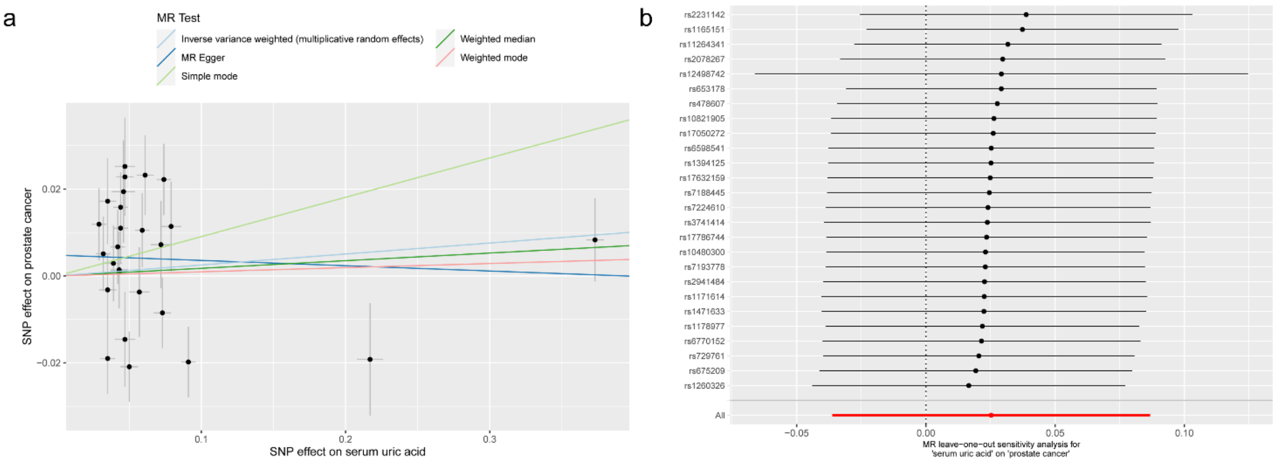

Supplementary Figure 5. Scatter plot and leave-one-out plot of the estimated effect of serum uric acid levels on risk of prostate cancer. (a) scatter plot; (b) leave-one-out plot.

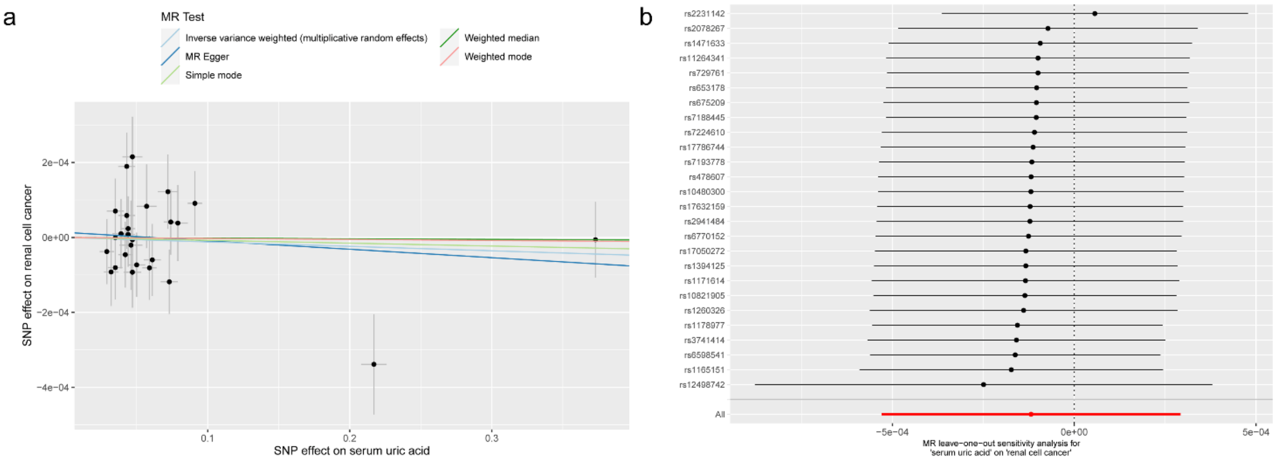

Supplementary Figure 6. Scatter plot and leave-one-out plot of the estimated effect of serum uric acid levels on risk of renal cell cancer. (a) scatter plot; (b) leave-one-out plot.

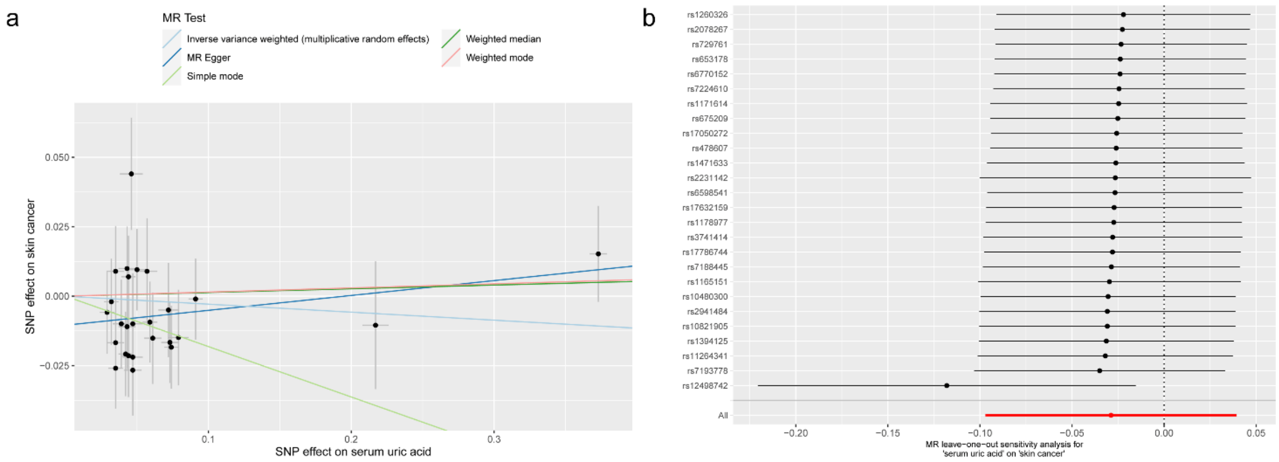

Supplementary Figure 7. Scatter plot and leave-one-out plot of the estimated effect of serum uric acid levels on risk of skin cancer. (a) scatter plot; (b) leave-one-out plot.

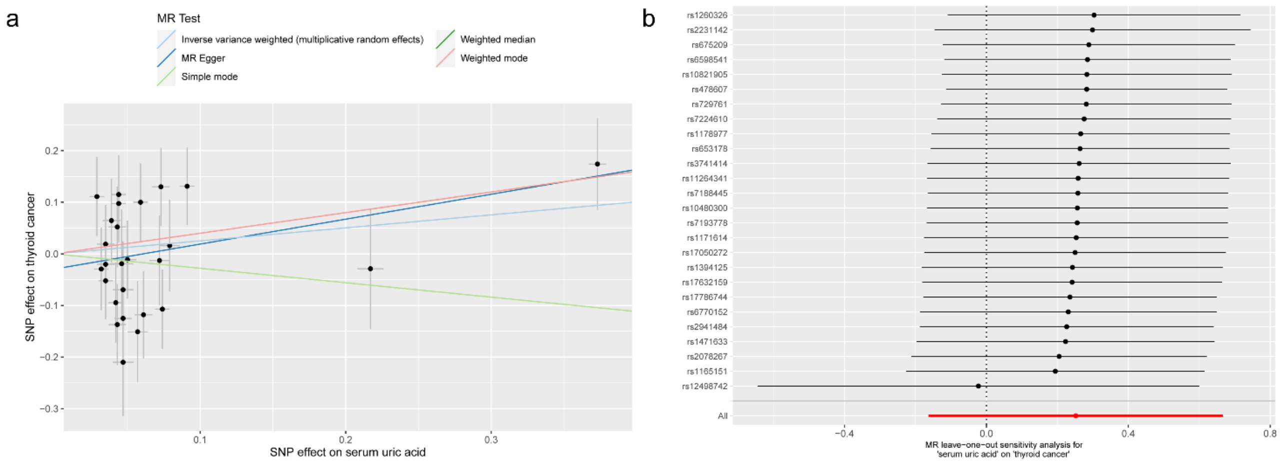

Supplementary Figure 8. Scatter plot and leave-one-out plot of the estimated effect of serum uric acid levels on risk of thyroid cancer. (a) scatter plot; (b) leave-one-out plot.

Cancer type	OR (95% CI)	P value
Bladder cancer	1.001(1.000-1.001)	0.021
Breast cancer	0.917(0.816-1.031)	0.147
Colorectal cancer	0.783(0.625-0.980)	0.033
Lung cancer	1.000(0.999-1.000)	0.437
Prostate cancer	1.030(0.936-1.133)	0.549
Renal cell cancer	1.000(0.999-1.000)	0.437
Skin cancer	0.889(0.809-0.976)	0.014
Thyroid cancer	0.978(0.525-1.822)	0.944

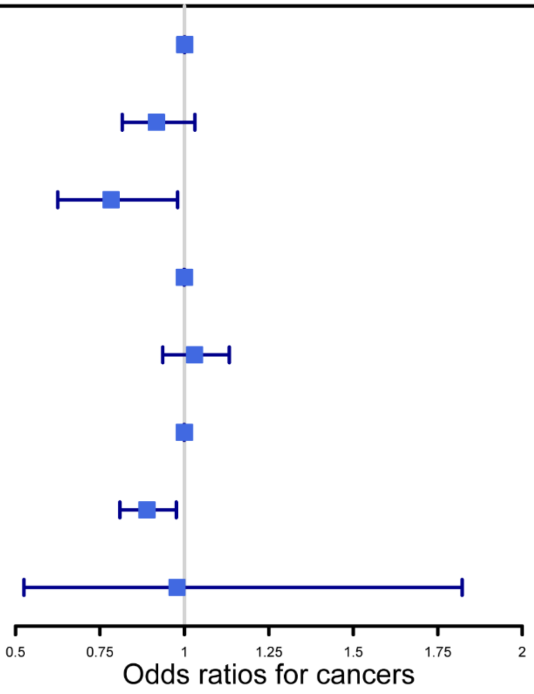

Supplementary Figure 9. Primary results of the causal associations between serum uric acid levels and cancer risk by random effects inverse variance weighted method after exclusion of rs12498742. OR, odds ratio; IVW, inverse variant weighted; CI, confidence interval.

Supplementary Table 1. Characteristics of SNPs included in the two-sample Mendelian randomization analysis.

SNP[a]	Chr[b]	Positon	Gene	EA[c]	OA[d]	EAF[e]	Beta	SE[f]	P value	F-statistic
rs11264341	1	155151493	TRIM46	T	C	0.43	-0.050	0.006	6.20E-19	69.40
rs1471633	1	145723739	PDZK1	A	C	0.46	0.059	0.005	1.20E-29	139.06
rs1260326	2	27730940	GCKR	T	C	0.41	0.074	0.005	1.20E-44	218.60
rs17050272	2	121306440	INHBB	A	G	0.43	0.035	0.006	1.60E-10	34.02
rs6770152	3	53100214	SFMBT1	T	G	0.58	-0.044	0.005	2.60E-16	77.38
rs12498742	4	9944052	SLC2A9	A	G	0.77	0.373	0.006	0[g]	3733.82
rs2231142	4	89052323	ABCG2	T	G	0.11	0.217	0.009	1.00E-134	57.28
rs17632159	5	72431482	TMEM171	C	G	0.31	-0.039	0.006	3.50E-11	42.23
rs1165151	6	25821616	SLC17A1	T	G	0.47	-0.091	0.005	7.00E-70	330.24
rs675209	6	7102084	RREB1	T	C	0.27	0.061	0.006	1.30E-23	103.26
rs729761	6	43804571	VEGFA	T	G	0.30	-0.047	0.006	8.00E-16	61.33
rs10480300	7	151406005	PRKAG2	T	C	0.28	0.035	0.006	4.10E-09	34.02
rs1178977	7	72857049	BAZ1B	A	G	0.81	0.047	0.007	1.20E-12	45.06
rs17786744	8	23777006	STC1	A	G	0.58	-0.029	0.005	1.40E-08	33.63
rs2941484	8	76478768	HNF4G	T	C	0.44	0.044	0.005	4.40E-17	77.38
rs10821905	10	52646093	A1CF	A	G	0.18	0.057	0.007	7.40E-17	66.26
rs1171614	10	61469538	SLC16A9	T	C	0.22	-0.079	0.007	2.30E-28	127.22
rs2078267	11	64334114	SLC22A11	T	C	0.51	-0.073	0.006	9.40E-38	147.83
rs478607	11	64478063	NRXN2	A	G	0.84	-0.047	0.007	4.40E-11	45.06
rs3741414	12	57844049	INHBC	T	C	0.24	-0.072	0.007	2.20E-25	105.69
rs653178	12	112007756	ATXN2	T	C	0.51	-0.035	0.005	7.20E-12	48.98
rs1394125	15	76158983	UBE2Q2	A	G	0.34	0.0430	0.006	2.50E-13	51.34

rs6598541	15	99271135	IGF1R	A	G	0.36	0.0430	0.006	4.80E-15	51.34
rs7188445	16	79734987	MAF	A	G	0.33	-0.032	0.005	1.60E-09	40.94
rs7193778	16	69563890	NFAT5	T	C	0.86	-0.046	0.008	8.20E-10	30.05
rs7224610	17	53364788	HLF	A	C	0.58	-0.042	0.005	5.40E-17	70.51

[a] SNP, single-nucleotide polymorphism
[b] Chr, Chromosome
[c] EA, Effect allele
[d] OA, Other allele
[e] EAF, Effect allele frequency
[f] SE, Standard error
[g] P value $< 1 \times 10^{-700}$

Supplementary Table 2. Five additional Mendelian randomization method for estimation of the causal associations between serum uric acid levels and cancer risk.

Cancer type	IVW (fixed-effect)		MR-Egger		Weighted median		Simple mode		Weighted mode	
	OR[a] (95% CI[b])	P value	OR (95% CI)	P value	OR (95% CI)	P value	OR (95% CI)	P value	OR (95% CI)	P value
Bladder cancer	1.000 (0.999-1.000)	0.415	1.000 (0.999-1.000)	0.343	1.000 (0.999-1.001)	0.813	1.001 (0.999-1.002)	0.444	1.000 (0.999-1.001)	0.905
Breast cancer	0.985 (0.939-1.034)	0.544	1.019 (0.912-1.139)	0.738	1.026 (0.963-1.093)	0.428	1.008 (0.850-1.195)	0.931	1.008 (0.949-1.070)	0.806
Colorectal cancer	0.905 (0.819-1.000)	0.051	1.050 (0.851-1.291)	0.654	1.008 (0.890-1.142)	0.900	0.977 (0.745-1.282)	0.870	0.994 (0.884-1.118)	0.923
Lung cancer	1.000 (0.999-1.000)	0.919	1.000 (0.999-1.000)	0.999	1.000 (1.000-1.001)	0.997	1.000 (0.999-1.001)	0.549	1.000 (0.999-1.000)	0.870
Prostate cancer	1.026 (0.987-1.065)	0.193	0.988 (0.906-1.078)	0.786	1.018 (0.971-1.067)	0.468	1.095 (0.871-1.375)	0.444	1.010 (0.961-1.061)	0.710
Renal cell cancer	1.000 (0.999-1.000)	0.559	1.000 (0.999-1.000)	0.467	0.999 (0.998-1.000)	0.946	1.000 (0.999-1.001)	0.917	1.000 (0.999-1.000)	0.920
Skin cancer	0.972 (0.908-1.040)	0.406	1.055 (0.957-1.163)	0.290	1.013 (0.931-1.103)	0.757	0.834 (0.669-1.040)	0.119	1.015 (0.933-1.104)	0.731
Thyroid cancer	1.287 (0.907-1.824)	0.157	1.620 (0.897-2.926)	0.122	1.491 (0.947-2.347)	0.084	0.756 (0.196-2.922)	0.689	1.492 (0.957-2.326)	0.090

[a] OR, Odds ratios

[b] CI, Confidence interval

Table S3. MR-Egger regression for pleiotropy assessment for effect of serum uric acid levels on cancer risk.

Cancer type	26 SNPs			6 SNPs		
	Intercept	SE[a]	P value	Intercept	SE	P value
Bladder cancer	7.53E-05	3.49E-05	0.05	6.40E-05	6.41E-05	0.38
Breast cancer	-4.46E-03	5.24E-03	0.40	1.05E-02	7.54E-03	0.24
Colorectal cancer	-1.88E-02	9.69E-03	0.06	5.50E-04	1.21E-02	0.97
Lung cancer	1.68E-06	1.72E-05	0.92	1.57E-06	3.15E-05	0.93
Prostate cancer	4.79E-03	4.04E-03	0.25	4.26E-03	4.59E-03	0.41
Renal cell cancer	1.35E-05	2.75E-05	0.63	-4.99E-05	4.81E-05	0.36
Skin cancer	-1.04E-02	4.49E-03	0.03	-1.36E-02	8.25E-03	0.17
Thyroid cancer	-2.93E-02	2.74E-02	0.29	4.33E-02	4.88E-02	0.43

[a] SE, Standard error

Table S4. Heterogeneity test for the primary results in the two-sample Mendelian randomization analysis.

Cancer type	26 SNPs		6 SNPs	
	**Q value**	**P value**	**Q value**	**P value**
Bladder cancer	16.48	7.74E-01	1.84	8.70E-01
Breast cancer	66.75	1.15E-05	9.86	7.90E-02
Colorectal cancer	60.27	9.60E-05	1.51	9.12E-01
Lung cancer	9.99	9.97E-01	2.07	8.40E-01
Prostate cancer	65.47	1.77E-05	4.78	4.43E-01
Renal cell cancer	26.71	3.70E-01	2.79	7.32E-01
Skin cancer	25.22	4.50E-01	4.90	4.28E-01
Thyroid cancer	35.34	8.23E-02	6.37	2.72E-01

Table S5. Characteristics of SNPs exclusively associated with serum uric acid levels

SNP[a]	Chr[b]	Positon	Gene	EA[c]	OA[d]	EAF[e]	Beta	SE[f]	P value	F-statistic
rs1471633	1	145723739	PDZK1	A	C	0.46	0.059	0.005	1.20E-29	139.06
rs12498742	4	9944052	SLC2A9	A	G	0.77	0.373	0.006	0[g]	3733.82
rs17632159	5	72431482	TMEM171	C	G	0.31	-0.039	0.006	3.50E-11	42.23
rs2941484	8	76478768	HNF4G	T	C	0.44	0.044	0.005	4.40E-17	77.38
rs2078267	11	64334114	SLC22A11	T	C	0.51	-0.073	0.006	9.40E-38	147.83
rs7224610	17	53364788	HLF	A	C	0.58	-0.042	0.005	5.40E-17	70.51

[a] SNP, single-nucleotide polymorphism
[b] Chr, Chromosome
[c] EA, Effect allele
[d] OA, Other allele
[e] EAF, Effect allele frequency
[f] SE, Standard error
[g] P value $< 1 \times 10^{-700}$

Table S6. Results of the causal associations between serum uric acid levels and cancer risk by IVW method using 6 SNPs.

Cancer type	OR[a]	95% CI[b]	P value
Bladder cancer	1.000	0.999-1.001	0.839
Breast cancer	1.055	0.971-1.148	0.207
Colorectal cancer	1.016	0.897-1.151	0.803
Lung cancer	1.000	0.999-1.001	0.962
Prostate cancer	1.027	0.980-1.077	0.270
Renal cell cancer	1.000	0.999-1.000	0.574
Skin cancer	1.013	0.646-1.055	0.126
Thyroid cancer	1.777	1.085-2.911	0.022

[a] OR, Odds ratios
[b] CI, Confidence interval
